# Female risk-adjusted survival advantage after injuries caused by falls, traffic or assault: a nationwide 11-year study

**DOI:** 10.1186/s13049-019-0597-3

**Published:** 2019-03-15

**Authors:** Robert Larsen, Denise Bäckström, Mats Fredrikson, Ingrid Steinvall, Rolf Gedeborg, Folke Sjoberg

**Affiliations:** 1 Department of Clinical and Experimental Medicine, Linkoping University, Linkoping, Sweden; 2 Department of Anaesthesiology and Intensive Care, and Department of Medical and Health Sciences, Linkoping University, S-58185 Linkoping, Sweden; 3 Life Regiment Hussars, K3 Karlsborg, Sweden; 4 Department of Hand Surgery, Plastic Surgery and Burns, and Department of Clinical and Experimental Medicine, Linkoping University, Linkoping, Sweden; 5 Department of Surgical Sciences, Anaesthesiology and Intensive Care, Uppsala University, Uppsala, Sweden

**Keywords:** Risk-adjusted mortality, ICISS, Trauma, Injury, Nationwide, Epidemiological

## Abstract

**Background:**

A female survival advantage after injury has been observed, and animal models of trauma have suggested either hormonal or genetic mechanisms as component causes. Our aim was to compare age and risk-adjusted sex-related mortality in hospital for the three most common mechanisms of injury in relation to hormonal effects as seen by age.

**Methods:**

All hospital admissions for injury in Sweden during the period 2001–2011 were retrieved from the National Patient Registry and linked to the Cause of Death Registry. The International Classification of Diseases Injury Severity Score (ICISS) was used to adjust for injury severity, and the Charlson Comorbidity Index to adjust for comorbidity. Age categories (0–14, 15–50, and ≥ 51 years) were used to represent pre-menarche, reproductive and post- menopausal women.

**Results:**

Women had overall a survival benefit (OR 0.51; 95% CI 0.50 to 0.53) after adjustment for injury severity and comorbidity. A similar pattern was seen across the age categories (0–14 years OR 0.56 (95% CI 0.25 to 1.25), 15–50 years OR 0.70 (95% CI 0.57 to 0.87), and ≥ 51 years OR 0.49 (95% CI 0.48 to 0.51)).

**Conclusion:**

In this 11-year population-based study we found no support for an oestrogen-related mechanism to explain the survival advantage for females compared to males following hospitalisation for injury.

**Electronic supplementary material:**

The online version of this article (10.1186/s13049-019-0597-3) contains supplementary material, which is available to authorized users.

## Introduction

A female survival advantage is well known [1–5], and is not restricted to particular regions or ethnicities. The magnitude of this difference is substantial, e.g. Japanese women outlive Japanese men by six years [6]. But it is interesting that it has not been convincingly reflected in the outcomes of medical diseases, though the same female survival advantage has been shown in models of trauma and sepsis in animals [7, 8]. Knowledge of the underlying mechanism of a female survival benefit are important as it may provide clues as to improve trauma care outcomes.

In clinical studies of the outcome of injury the results regarding the potential impact of sex have been contradictory, in that some have shown a female advantage, some a disadvantage, and some no difference [9–17]. A national study on Swedish intensive care unit patients [18] showed similar survival rates for men and women, but male patients had significantly more interventions. Recent studies in trauma have suggested a female survival advantage [19], also after adjustment for age and coexisting diseases [20].

Two physiological mechanisms were suggested in models of trauma in animals to explain such female survival advantage: hormonal response [19] to injury, or genetic advantage in the physiological response to injury [21]. Differences in health care could also potentially contribute to a difference in outcome. A study by Gomez found that a lower proportion of female patients, compared to males, were transferred to trauma centres [22].

Using age as a surrogate marker for female sex hormonal levels, it might be possible to differentiate between hormonal effects in a retrospective registry study. In addition, we can adjust for comorbidity, and stratify the mechanisms of trauma into three major well-defined subgroups of injuries, in order to improve comparison and identify independent associations between sex and survival following trauma.

Our main hypothesis was that oestrogen is protective and the main contributing factor for female survival advantage in trauma. The hypothesis is further supported by a clinical trial in which the effect on female trauma patients is examined as a sub analysis [17].

## Methods

### Patients studied

All hospital admissions for falls, road traffic crashes, or assault during the years 2001–2011 in Sweden were retrieved from the National Patient Registry (which covers all admissions to Swedish hospitals since 1987), and the Cause of Death Registry (which covers all deaths of Swedish citizens). Patients who died before reaching hospital or who had injuries that did not require hospital admission were not included. For patients who were transferred between departments during treatment for the same injury we used the date of admission and diagnoses from the first record and the date of discharge from the last admission. These records were linked to all records in the Cause of Death Registry that had “injury” as the main cause of death (V01-Y98.9) using the unique personal identification number given to everyone who lives permanently in Sweden. Records from which information on age, sex, date of admission, or mechanism of injury was missing were excluded from the analyses [20].

**Fig. 1:**
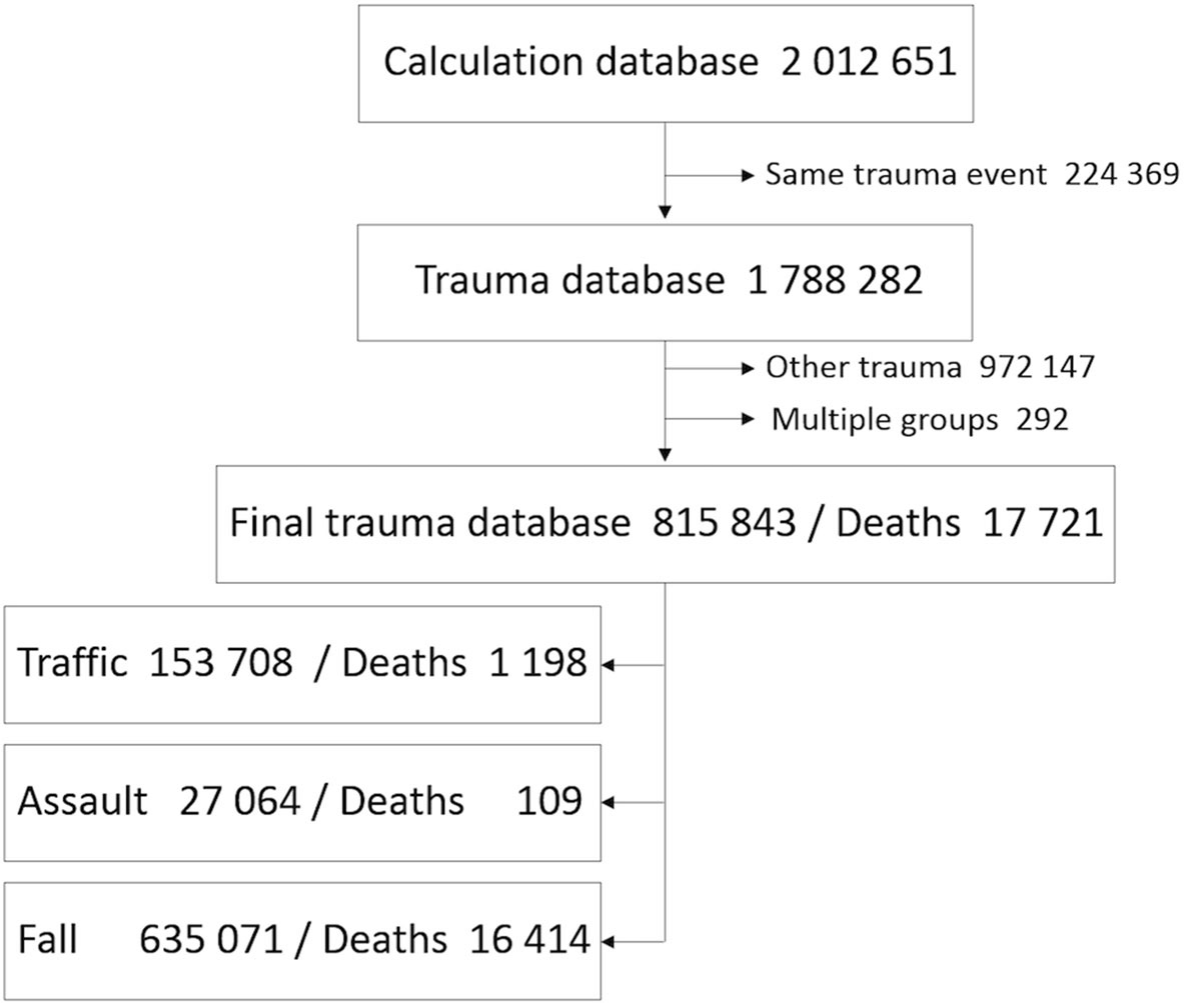
Flowchart showing selection of patients. Calculation database: data from National Patient Registry and Cause of Death Registry combined. Trauma database: removal of duplicates (224369) in same trauma event. Final trauma database: removal of other mechanisms than fall, traffic and assault, and of the 292 trauma recordings for patients who were classified as having more than one mechanism. The Final trauma database was used for all calculations

Those in which the cause of injury was “fall” (W00-W19), “traffic incident” (V01-V99), or “assault” (X85-Y09) were then selected for further study (Fig. 1). A few observations (*n* = 292, 0.036%) were classified in more than one group, and they were excluded.

### Identification of death and 30-day mortality

Data from the Causes of Death Registry were available until 31 December 2012, which allowed at least 12 months’ follow-up after the date of admission to hospital (considered to be the index date of the injury). Thirty-day mortality was calculated to include most of the patients who died as a direct result of the injury, and to exclude those who died mainly of other causes.

### Severity of injury

The Injury Severity Score (ISS) has long been regarded as the standard measure of severity of injury. In 1996 Osler et al. developed a score based on International Classification of Disease (ICD)-9 hospital discharge diagnoses (ICISS) [23], so that they could use large administrative databases with diagnostic codes. Later studies showed that ICISS calculated from ICD10 was superior and allowed a more accurate estimate of the severity of injury [24].

Duplicate ICD10 codes in the National Patient Registry were omitted before calculating the ICISS score. The diagnosis-specific probabilities were estimated using the main injury diagnosis codes and up to nine secondary codes.

We used ICISS as the risk-adjustment for 30-day mortality counting from the first hospital admission.

### Comorbidity

The Charlson Comorbidity Index (CCI) was calculated using the weighted scale as described in the original paper [25] and the ICD-codes from Christensen et al. [26].

### Definition of the hormonal subgroup

The age group from 15 to 50 years was used to identify postmenarchal and premenopausal women [27]. Sensitivity analyses were performed comparing the age groups 0–10, 20–40, and 60-, in order to evaluate the potential impact of misclassification due to individual variation in the age for menarche and menopause with same result but lower precision due to lower numbers (data not shown).

### Statistical analyses

Logistic regression models with ICISS, CCI, age (years) and sex, were used to estimate the association between sex and 30-day mortality. Numerical variables were used as linear effects without transformation in the models. The discrimination, i.e. the model’s ability to separate those who died from those who survived, was measured by the area under the receiver operating characteristic curve (AUC). Probabilities of less than 0.05 were accepted as significant.

We used the statistics software Stata (Stata Corp LP 2011–15, Stata version 12–15, College Station, TX, USA) for data management and statistical analyses.

## Results

The study population consisted of 815,843 hospital admissions for the three causes of injury (Fig. 1). Fifty-four percent were female. The mean age was 58 (range 0–111) years, and women were significantly older than men. Crude 30-day mortality was 2.2%, with a lower crude mortality for women (*p* < 0.001) with a difference of 0.3% between the groups (men 2.3% and women 2.0%). Median ICISS decreased with age group but no statistical difference between the sexes were able to be detected. Thirty-day mortality increased with age group, and male sex was over-represented in crude mortality throughout the groups (Table 1).

**Table 1:** Description of the study population characteristics

		All	Male	Female
No of patients, count (%)		815,843 (100)	373,811 (46)	442,032 (54)
Deaths, count (%)		17,721 (100)	8679 (49)	9042 (51)
Age in years, mean (SD)		58 (29)	49 (28)	65 (27)
Age group				
0–14 years “Premenarche”, count (%)	Total	95,135 (100)	57,506 (60)	37,629 (40)
Count, (% of the number above)	Deaths	33 (0.03)	23 (0.04)	10 (0.03)
	Median ICISS	0.98	0.98	0.98
15–50 years “Reproductive”, count (%)	Total	195,582 (100)	127,251 (65)	68,331 (35)
Count, (% of the number above)	Deaths	591 (0.30)	459 (0.36)	132 (0.19)
	Median ICISS	0.97	0.97	0.98
Over 50 years “Postmenopausal”, count (%)	Total	525,129 (100)	189,054 (36)	336,075 (64)
Count, (% of the number above)	Deaths	17,097 (3.26)	8197 (4.34)	8900 (2.65)
	Median ICISS	0.93	0.94	0.93

*Abbreviation:* *SD* Standard deviation

**Fig. 2:**
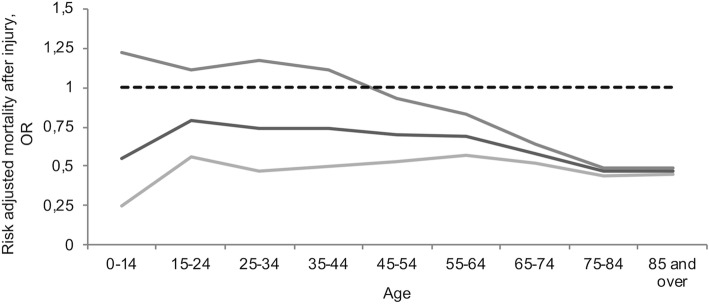
Graph showing female survival benefit with 95% CI compared to male by age group. Solid black line Female Risk-Adjusted Survival Advantage. Solid grey lines 95% CI of Female Risk-Adjusted Survival Advantage. Dotted black line for comparison and male reference

The main result was that women overall in the entire study population had a survival benefit (OR 0.51, 95% CI 0.50 to 0.53) after adjustment for injury severity and comorbidity. Subgroup analysis showed that the pattern was similar across the three age groups. When doing separate analyses for the severely injured (ICISS<=0.85) the same pattern were obvious (OR 0.74, 95% CI 0.69 to 0.79). In premenarche (OR 0.56; 95% CI 0.25 to 1.25), during reproductive age (OR 0.70; 95% CI 0.57 to 0.87), and postmenopausal (OR 0.49; 95% CI 0.48 to 0.51), although precision in the premenarche risk estimate was low due to the relatively few deaths (Table 2). When we assessed smaller groups based on age to avoid misclassification of the hormonal concentration, the same trends were evident, but there was more uncertainty in the estimated associations (Fig. 2). The mortality increased exponentially with age (Table 3).

**Table 2:** Logistic regression for 30-day mortality including subgroup analysis

	OR	p	95% CI	R^2a^	AUC
Total		< 0.001		0.215	0.876
ICISS	< 0.001	< 0.001	< 0.001 to < 0.001		
CCI	1.269	< 0.001	1.253 to 1.283		
Female	0.512	< 0.001	0.496 to 0.529		
Age (years)	1.075	< 0.001	1.074 to 1.077		
Constant	10.422	< 0.001	8.706 to 12.475		
Pre menarche		< 0.001		0.255	0.894
ICISS	< 0.001	< 0.001	< 0.001 to < 0.001		
CCI	1.125	0.9270	0.092 to 13.832		
Female	0.563	0.1580	0.254 to 1.249		
Age (years)	0.956	0.2390	0.887 to 1.030		
Constant	4392.84	< 0.001	510.493 to 37,800.8		
Reproductive		< 0.001		0.294	0.931
ICISS	< 0.001	< 0.001	< 0.001 to < 0.001		
CCI	1.452	< 0.001	1.238 to 1.703		
Female	0.704	0.0010	0.573 to 0.865		
Age (years)	1.009	0.0300	1.001 to 1.017		
Constant	150.692	< 0.001	93.326 to 243.320		
Menopause		< 0.001		0.156	0.820
ICISS	< 0.001	< 0.001	< 0.001 to < 0.001		
CCI	1.277	< 0.001	1.262 to 1.292		
Female	0.495	< 0.001	0.479 to 0.511		
Age (years)	1.090	< 0.001	1.088 to 1.092		
Constant	2.861	< 0.001	2.293 to 3.570		

^a^Pseudo R^2^

*Abbreviations:* *AUC* Area under the curve, *CCI* Charlson comorbidity index, *CI* Confidence interval, *ICISS* International classification of disease injury severity score, *OR* Odds ratio, *p* Probability, *R2* Coefficient of determination

**Table 3:** Logistic regression for 30-day mortality by age group and sex

		Coefficient	p	OR	95% CI
ICISS		−10.6684	< 0.001		
Age group	Sex				
0–14	Women	−0.1074	0.551	0.898	0.631 to 1.279
15–25	Men	0.8001	< 0.001	2.226	1.743 to 2.843
15–25	Women	0.6840	< 0.001	1.982	1.515 to 2.593
26–35	Men	1.0752	< 0.001	2.931	2.284 to 3.760
26–35	Women	0.9637	< 0.001	2.621	1.980 to 3.470
36–45	Men	1.2114	< 0.001	3.358	2.633 to 4.283
36–45	Women	1.1070	< 0.001	3.025	2.321 to 3.943
46–55	Men	1.7065	< 0.001	5.510	4.367 to 6.951
46–55	Women	1.4370	< 0.001	4.208	3.285 to 5.391
56–65	Men	2.1299	< 0.001	8.414	6.710 to 10.550
56–65	Women	1.8096	< 0.001	6.108	4.836 to 7.715
66–75	Men	2.8235	< 0.001	16.837	13.476 to 21.035
66–75	Women	2.3141	< 0.001	10.116	8.078 to 12.668
> 76	Men	3.9751	< 0.001	53.255	42.774 to 66.303
> 76	Women	3.3639	< 0.001	28.902	23.217 to 35.978

Men age younger than 15 as reference.

*Abbreviations:* *CI* Confidence interval, *ICISS* International classification of disease injury severity score, *OR* Odds ratio, *p* probability

In analyses within the age subgroups, female sex was still associated with a survival advantage, although these estimates had low precision in the younger age groups due to relatively few deaths (Table 3). A separate analysis based on mechanism was included in Additional file 1. It is notable that there was no female survival benefit in the premenarche assault group.

C-statistic analysis showed high values for the AUC in all groups, with the highest value in the reproductive group (Table 2). 

## Discussion

The aim of this study was to estimate the association between sex and short-term survival following injury, within age categories reflecting levels of female sexual hormones, and adjusted for injury severity and comorbidity. The study results suggest that the female survival advantage in the predominant causes of injury in Sweden (road traffic, fall, and assault) is not more pronounced in the age range where the levels of female sex hormones are expected to be naturally higher. This suggests that hormonal levels do not mainly explain the female survival advantage following injury.

The survival advantage for females appears consistent across the age categories. If the hormonal component is of major importance, an added survival advantage during the hormone-producing years of life would have been expected. If anything, we observed a somewhat attenuated survival advantage for females during that age span. This further supports that other mechanisms than levels of female sexual hormones explain this difference.

Sensitivity analyses using more restrictive age groups, to reduce potential misclassification of menarche and menopause, provided similar estimates but with lower precision. These results further support the conclusions.

### Strengths of the study

The population-based design with reliable follow-up based on the exact link of personal records with the Cause of Death Registry, even after hospital discharge, and accurate estimates of the severity of injury are notable strengths. The ICISS provides an accurate estimation of the severity of injury [24]. The quality of the coding of injuries in the Swedish National Patient Registry has previously been validated, and is accurate to the fourth position of the code [28]. It has been shown in a comparison among eight countries that the similarities in ICISS are substantial, which further strengthens our results [29]. Previous studies in Sweden have not shown a resource allocation that predisposed to female survival, but have shown that more resources were allocated to men, which argues against such factors being of major importance for differences in survival between men and women [18].

### Limitations of the study

The relatively low number of deaths in the younger groups limits the precision of estimated associations. Another limitation is that we did not include prehospital data in our study, but as previous studies showed no difference in risk-adjusted survival between prehospital and in-hospital mortality [30], adding prehospital data could have increased the precision, but the main results would likely not be different. Another limitation is that the data is not adjusted for interventions (not surgical nor medical) and this is a limitation of the current study. Interventions should be a part of the adjustment in further studies. Another limitation is the expected misclassification of hormonal levels, e.g. from postmenopausal hormonal replacement therapy. This could be further examined in the Swedish setting today by using the Medical Prescription Registry. The notable lack of female survival benefit in the premenarche assault group could be explained by the low number of deceased (2 patients) and both of them were male.

### Strengths, weaknesses, and important differences in results compared with other studies

It has previously been shown that Sweden compares well with other countries in the recording and treatment of trauma [29]. Even though coding-errors in ICD-10 are common, the consequences for estimates of the severity of injury are claimed to be minor in most cases [28]. To our knowledge this is the first population-based nationwide study that has investigated ICISS risk-adjusted 30-day survival by sex in patients admitted to hospital. Another strength is that healthcare in Sweden is publicly financed and not dependent on the patient’s funds or insurance, which further supports the view that our model is estimating the physiological effect rather than financial or administrative effects of healthcare.

### Meaning of the study: Possible explanations and implications for clinicians and policymakers

This study has shown a risk-adjusted, 30-day, survival advantage after trauma for women compared with men but without major differences across age categories representing expected levels of female sexual hormones. These results do not support the idea that trauma patients should be given oestrogen [31].

### Unanswered questions and future research

In our analyses, sex was a significant risk factor for mortality even after adjustment for injury mechanism and severity. While specific health-care interventions should be evaluated [22], it is also important to try to understand the mechanism behind the observed association between sex and mortality after injury. The higher mortality for men may suggest that men received suboptimal treatment, but previous studies, if anything, support a male advantage in this respect [18].

## Conclusion

In this population-based study over an 11-year period we did not after adjustments for injury severity, age, and co-morbidity find any support for a hormonal effect (oestrogen) explaining a female survival benefit.

## Additional file

Additional file 1: Supplemental digital content. (DOCX 34 kb)

## Abbreviations

AUC: Area under the curve
CCI: Charlson comorbidity index
CI: Confidence interval
ICD-9/10: International classification of disease version 9/10
ICISS: International classification of disease injury severity score
ISS: Injury severity score
OR: Odds ratio
R^2^: Coefficient of determination
SD: Standard deviation
