# Supplementary material for: Female risk-adjusted survival advantage after injuries caused by falls, traffic or assault: a nationwide 11-year study
Source: Scand J Trauma Resusc Emerg Med. 2019 Mar 15;27:24. doi: 10.1186/s13049-019-0597-3 (PMC6419337; doi:10.1186/s13049-019-0597-3)
Supplement: Supplementary file 1 — Supplemental digital content. (DOCX 34 kb) [file 13049_2019_597_MOESM1_ESM.docx]

Additional file 1

**Supplemental Digital Content**

| **Fall** | | | | | | | |
| --- | --- | --- | --- | --- | --- | --- | --- |
|  | | OR | p | 95% CI | R^2^* | n |  |
| **Total** | |  |  |  | 0.196 | 635 071 |  |
|  | ICISS | <0.001 | <0.001 | <0.001 to <0.001 |  |  |  |
|  | CCI | 1.274 | <0.001 | 1.258 to 1.290 |  |  |  |
|  | Female | 0.490 | <0.001 | 0.469 to 0.502 |  |  |  |
|  | Age (years) | 1.087 | <0.001 | 1.085 to 1.089 |  |  |  |
|  | Constant | 6.095 | <0.001 | 4.785 to 7.763 |  |  |  |
| **Pre menarche** | |  |  |  | 0.234 | 69 649 |  |
|  | ICISS | <0.001 | <0.001 | <0.001 to <0.001 |  |  |  |
|  | CCI | 1 | - | - |  |  |  |
|  | Female | 3.488 | 0.325 | 0.290 to 42.014 |  |  |  |
|  | Age (years) | 0.928 | 0.573 | 0.714 to 1.205 |  |  |  |
|  | Constant | 430.214 | 0.088 | 0.408 to 453366.7 |  |  |  |
| **Reproductive** | |  |  |  | 0.2547 | 87 278 |  |
|  | ICISS | <0.001 | <0.001 | <0.001 to <0.001 |  |  |  |
|  | CCI | 1.631 | <0.001 | 1.377 to 1.933 |  |  |  |
|  | Female | 0.384 | <0.001 | 0.225 to 0.654 |  |  |  |
|  | Age (years) | 1.029 | 0.003 | 1.010 to 1.049 |  |  |  |
|  | Constant | 198.616 | <0.001 | 58.383 to 675.679 |  |  |  |
| **Menopause** | |  |  |  | 0.148 | 477 631 |  |
|  | ICISS | <0.001 | <0.001 | <0.001 to <0.001 |  |  |  |
|  | CCI | 1.275 | <0.001 | 1.260 to 1.291 |  |  |  |
|  | Female | 0.484 | <0.001 | 0.468 to 0.500 |  |  |  |
|  | Age (years) | 1.089 | <0.001 | 1.087 to 1.091 |  |  |  |
|  | Constant | 4.877 | <0.001 | 3.779 to 6.300 |  |  |  |
| * Pseudo R^2^ | | | | | | | |

| **Traffic** | | | | | | | |
| --- | --- | --- | --- | --- | --- | --- | --- |
|  | | OR | p | 95% CI | R^2^* | n |  |
| **Total** | |  |  |  | 0.307 | 153 708 |  |
|  | ICISS | <0.001 | <0.001 | <0.001 to <0.001 |  |  |  |
|  | CCI | 1.224 | <0.001 | 1.123 to 1.335 |  |  |  |
|  | Female | 0.708 | <0.001 | 0.619 to 0.809 |  |  |  |
|  | Age (years) | 1.037 | <0.001 | 1.034 to 1.040 |  |  |  |
|  | Constant | 31.444 | <0.001 | 22.207 to 44.524 |  |  |  |
| **Pre menarche** | |  |  |  | 0.269 | 23 966 |  |
|  | ICISS | <0.001 | <0.001 | <0.001 to <0.001 |  |  |  |
|  | CCI | 2.266 | 0.171 | 0.703 to 7.300 |  |  |  |
|  | Female | 0.479 | 0.108 | 0.196 to 1.175 |  |  |  |
|  | Age (years) | 0.864 | 0.001 | 0.790 to 0.944 |  |  |  |
|  | Constant | 4890.553 | <0.001 | 423.961 to 56414.460 |  |  |  |
| **Reproductive** | |  |  |  | 0.301 | 86 018 |  |
|  | ICISS | <0.001 | <0.001 | <0.001 to <0.001 |  |  |  |
|  | CCI | 1.228 | 0.353 | 0.796 to 1.895 |  |  |  |
|  | Female | 0.807 | 0.077 | 0.636 to 1.023 |  |  |  |
|  | Age (years) | 1.005 | 0.298 | 0.995 to 1.015 |  |  |  |
|  | Constant | 113.453 | <0.001 | 63.810 to 201.737 |  |  |  |
| **Menopause** | |  |  |  | 0.265 | 43 724 |  |
|  | ICISS | <0.001 | <0.001 | <0.001 to <0.001 |  |  |  |
|  | CCI | 1.184 | <0.001 | 1.081 to 1.297 |  |  |  |
|  | Female | 0.650 | <0.001 | 0.551 to 0.767 |  |  |  |
|  | Age (years) | 1.069 | <0.001 | 1.061 to 1.077 |  |  |  |
|  | Constant | 2.621 | 0.003 | 1.381 to 4.974 |  |  |  |
| * Pseudo R^2^ | | | | | | | |

| **Assault** | | | | | | | |
| --- | --- | --- | --- | --- | --- | --- | --- |
|  | | OR | p | 95% CI | R^2^* | n |  |
| **Total** | |  |  |  | 0.218 | 27 064 |  |
|  | ICISS | <0.001 | <0.001 | <0.001 to <0.001 |  |  |  |
|  | CCI | 1.422 | 0.030 | 1.035 to 1.952 |  |  |  |
|  | Female | 0.737 | 0.242 | 0.441 to 1.230 |  |  |  |
|  | Age (years) | 1.038 | <0.001 | 1.027 to 1.050 |  |  |  |
|  | Constant | 20.624 | <0.001 | 6.545 to 64.990 |  |  |  |
| **Pre menarche** | |  |  |  | 0.123 | 619 |  |
|  | ICISS | <0.001 | 0.057 | <0.001 to 1.353 |  |  |  |
|  | CCI | 1 | - | - |  |  |  |
|  | Female | 1 | - | - |  |  |  |
|  | Age (years) | 0.949 | 0.678 | 0.743 to 1.213 |  |  |  |
|  | Constant | 4890.553 | <0.001 | 423.961 to 56414.460 |  |  |  |
| **Reproductive** | |  |  |  | 0.222 | 22 286 |  |
|  | ICISS | <0.001 | <0.001 | <0.001 to <0.001 |  |  |  |
|  | CCI | 1.142 | 0.719 | 0.555 to 2.351 |  |  |  |
|  | Female | 0.674 | 0.291 | 0.323 to 1.403 |  |  |  |
|  | Age (years) | 1.031 | 0.015 | 1.006 to 1.057 |  |  |  |
|  | Constant | 33.707 | <0.001 | 7.281 to 156.044 |  |  |  |
| **Menopause** | |  |  |  | 0.160 | 3 771 |  |
|  | ICISS | <0.001 | <0.001 | <0.001 to <0.001 |  |  |  |
|  | CCI | 1.548 | 0.016 | 1.084to 2.212 |  |  |  |
|  | Female | 0.788 | 0.535 | 0.372 to 1.672 |  |  |  |
|  | Age (years) | 1.051 | 0.001 | 1.020 to 1.083 |  |  |  |
|  | Constant | 5.024 | 0.257 | 0.309 to 81.760 |  |  |  |
| * Pseudo R^2^ | | | | | | | |
| *Abbreviations for Supplemental Digital Content:*  CCI - Charlson Comorbidity Index, CI - Confidence Interval, ICISS - International Classification of disease Injury Severity Score, n – number, OR - Odds Ratio, p - probability, R2 - Coefficient of determination | | | | | | | |
